# Composition of yoga-philosophy based mental traits (*Gunas*) in major psychiatric disorders: A trans-diagnostic approach

**DOI:** 10.3389/fpsyg.2023.1075060

**Published:** 2023-02-01

**Authors:** Hemant Bhargav, Najla Eiman, Nishitha Jasti, Pooja More, Vinod Kumar, Bharath Holla, Rashmi Arasappa, Naren P. Rao, Shivarama Varambally, B.N. Gangadhar, Matcheri S. Keshavan

**Affiliations:** ^1^Department of Integrative Medicine, National Institute of Mental Health and Neurosciences (NIMHANS), Bengaluru, Karnataka, India; ^2^Department of Psychiatry, National Institute of Mental Health and Neurosciences (NIMHANS), Bengaluru, Karnataka, India; ^3^Massachusetts Mental Health Center, Boston, MA, United States; ^4^Beth Israel Deaconess Medical Center, Harvard Medical School, Boston, MA, United States

**Keywords:** yoga-based personality, psychiatric disorders, mental attributes, *Guna*, cross-sectional, experimental study

## Abstract

Yoga philosophy includes the theory of *Tri-guna* (three mental traits): *sattva* (signifies a tendency to ‘goodness’), *rajas* (tendency towards ‘activity’), and *tamas* (tendency towards “inertia”). This cross-sectional study aimed to understand the differences in the expression of *gunas* in patients suffering from major psychiatric disorders (*n* = 113, 40 females) and age-gender-education-matched healthy controls (HCs; *n* = 113, 40 females). Patients were diagnosed by a psychiatrist using DSM 5 criteria and suffered from the following disorders: depression (*n* = 30), schizophrenia (SCZ; *n* = 28), obsessive–compulsive disorder (OCD; *n* = 23), anxiety (*n* = 16), and bipolar affective disorder (BPAD; *n* = 16). *Tri-gunas* were assessed using a validated tool (Vedic Personality Inventory) and symptoms were assessed using standard scales as per the diagnosis. Multi-variate analysis of variance (MANOVA) was used to assess the differences in *guna* scores between HCs and patients, and between patients with different diagnoses. A two-tailed Pearson correlation was performed between the *gunas* and psychometric scales. Results revealed that HCs had significantly higher *sattva* traits as compared to patients (except those with OCD). Each psychiatric diagnosis also showed a specific *guna* configuration: (1) Anxiety disorders and OCD: High *sattva-rajas*, low *tamas*; (2) Depression: High *sattva-tamas*, low *rajas*; (3) Psychotic disorders (SCZ/BPAD): High *tamo-rajas*, low *sattva*. Significant positive correlations were observed between *rajas* traits and anxiety/OC/positive psychotic symptoms, negative psychotic symptoms and *tamas* traits, and *sattva* traits and OC symptoms. This finding has clinical implications, both to develop ways of predicting outcomes of psychiatric disorders, as well as to develop psycho-therapeutic and lifestyle interventions targeting the *gunas*.

## 1. Introduction

Eastern philosophies, including that of psychological concepts and theories, have influenced Western thought since ancient times. Although theoretical attempts have been made to bridge the gap between Eastern and Western philosophies (Bobade and Khale, 2019; Venkatanagarajan and Kamalanabhan, 2019), very few experimental studies have attempted to understand the human psyche from both modern and traditional perspectives (Mulla and Krishnan, 2019; Xu et al., 2021). Studies report persistent disparity in primary mental healthcare of patients belonging to different ethnicity and culture (Illes et al., 2015). To eliminate this disparity and enhance patient-centered integrated care there is a need for research and training in trans-diagnostic approaches that aim at developing a link between western diagnostic tools and culturally responsive traditional assessments (Raguram et al., 2001; Illes et al., 2015).

Insights into understanding human nature as per Indian thought have been derived from ancient yogic scriptures, which mainly include the *Vedas, Upanishads*, *Patanjali’s Yoga Sutra* (P.Y.S.), and *Bhagavad Gita* (B.G.). Hindu philosophy has six different schools of philosophy, called *Darshanas*. The two major *darshanas* are *Samkhya* and *Yoga,* which explain the psychological attributes or personality of an individual. This is a dualistic philosophy that postulates two interdependent, simultaneously existing realities: the *purusha* (consciousness) and *prakriti* (nature and matter; P.Y.S. 4.34; Sedlmeier and Srinivas, 2016; Lauricella, 2021). *Purusha* has been described as the unchanging, attribute-less, and innermost core of the personality, which is omnipresent (universal consciousness), forming the basis for the existence of *prakriti*. *Prakriti*, on the other hand, includes everything that follows the law of change over time, whether it is physical or psychological (B.G. 13.20; Gambhirananda, 1984; Lauricella, 2021).

The psychological dimension of prakriti has been classified into three attributes depending on the way they manifest in human behavior. These psychological attributes of *prakriti* are called *gunas* in yoga philosophy (P.Y.S. 2.18). In the word *triguna*, “tri” stands for three, while *guna* stands for “subtle traits of nature.” The *trigunas* are named as follows: (1) *Sattva*, (2) *Rajas*, and (3) *Tamas* (B.G. 14.5; Srivastava, 2012; Datar and Murthy, 2019). *Sattva guna* represents the qualities of purity, goodness, well-being, control over senses, and attachment to happiness and knowledge. The *sattva guna* is the “quality of goodness.” When *sattva guna* is dominant, a person has an inherent desire to be good and caring (B.G. 14.6; Deshpande et al., 2009; Srivastava, 2012). *Rajas guna* is the manifestation of attachment to action and its results. Propelled by passion and desire, it leads to emotional attachment, impulsivity and a strong sense of doer-ship (B.G. 14.7). *Rajas guna* is the “active quality.” *Tamas guna*, on the contrary, is a tendency towards inertia, sleep, emotional bluntness, withdrawal from duties, and inflexibility and rigidity of ideas. It is the “quality of inertia” (B.G. 14.8; Gambhirananda, 1984; Srivastava, 2012). *Bhagavad Gita* also describes a transcendental personality trait called “*Gunatita*” which means ‘beyond *gunas*’. Constant focus of an individual with *Gunatita* personality trait is on identification with the consciousness *(purusha).* Such mind is characterized by high levels of cognitive flexibility with a meta-cognitive awareness about the inter-play of the above mentioned three *gunas* without attraction or aversion towards them (Bhagvad Gita 3.28 and 14.22; Gambhirananda, 1984).

Research on understanding psychopathology based on *yogic* concepts of *trigunas* has been scarce. A few cross-sectional studies comparing patients with specific psychiatric disorders and healthy controls have been performed. The disorders addressed were: (1) anxiety (*n* = 30 patients, 30 Healthy controls; Sharma et al., 2012), (2) depression (*n* = 20 patients, 20 healthy controls; Anoop Kumar and Balodhi, 2016), and (3) psychotic conditions (*n* = 15 patients with psychosis, 30 healthy controls; Lakshmi Bai et al., 1975). All these three preliminary studies revealed that patients had higher scores for *rajas* or *tamas* traits and lower *sattva* traits as compared to healthy controls. A recent cross-sectional study also demonstrated that yoga practitioners had higher *sattva* traits as compared to those who prefer physical exercise with a differential pattern of neuronal activation in areas of brain related to self-regulation and inhibitory control (Kaur et al., 2022). We also observed that higher *rajas* or *tamas* scores in healthy individuals are associated with higher perceived stress and lower life satisfaction (Sharma et al., 2021). A single-arm prospective study on 28 patients with opioid use disorder who were admitted in a rehabilitation center showed that it was possible to enhance *sattva* traits and reduce *tamas* traits by imparting a specific yoga-based lifestyle intervention of 4 weeks. Interestingly, this change in *guna* traits correlated with improvement in psycho-pathology (Devi et al., 2018). In our recent manuscript, we discussed the psychotherapeutic potential of yoga-philosophy and emphasized the need of assessing *gunas* in psychiatric patients for developing interventions targeted on *guna* modification (Bhide et al., 2021).

Thus, the present study was planned with following objectives: (1) understanding *guna* distribution in patients with major psychiatric illnesses (*n* = 113; anxiety disorder, depression, schizophrenia, obsessive compulsive disorder and bipolar affective disorder) in comparison to age, sex, and education-matched healthy controls (*n* = 113); (2) To observe the variability in *guna* patterns across different psychiatric diagnoses; (3) To understand the relationship between psychiatric symptoms (as assessed through standardized psychometric scales) and *guna* traits in patients. We hypothesized that: (1) patients with psychiatric disorders would have higher *rajas* and *tamas* traits and lesser *sattva* traits than healthy subjects; (2) patients with psychotic disorders would have higher *rajas* and *tamas* traits than patients with non-psychotic disorders; (3) symptoms of anxiety and positive psychotic symptoms would correlate positively with *rajas* traits, whereas symptoms of depression and negative psychotic symptoms would correlate with *tamas* traits.

## 2. Materials and methods

### 2.1. Subjects

One-hundred and thirteen adults (age mean ± SD: 31.5 ± 11.1 years, 40 females) with major psychiatric disorders (diagnosed by a psychiatrist as per DSM-5 based on clinical interview and corroborated by two independent psychiatrists) were recruited from in-patient and out-patient services of a tertiary mental health care hospital in Bengaluru, India. Likewise, 113 age-, sex -, and education-matched healthy controls (age mean ± SD: 30.4 ± 8.57 years, 40 females) were recruited from students and staff of nearby educational institutions and universities. They were screened using general health questionnaire (GHQ-12), and a clinical interview with a psychiatrist based on Mini International Neuropsychiatric Interview (MINI) Screen 7.0.2 for DSM-5. Table 1 provides demographic details of the participants.

**Table 1:** Demographic details of the healthy controls and patients with psychiatric disorders.

**SN**	**Variable name**	**Healthy controls (HCs)**	**Patients with common psychiatric disorders**	***p* Value (Independent sample *t*-test)** *
**1**	Age (years)	30.4 ± 8.57	31.52 ± 11.15	0.31
**2**	Gender	M = 73; *F* = 40	M = 73; F = 40	
**3**	Education (years)	16 ± 5.1	17 ± 4.2	0.11
**4**	Diagnosis and number of subjects	Healthy (*n* = 113) (GHQ-12 ≤ 3)	(a) Depression (*n* = 30); (b) Paranoid Schizophrenia (*n* = 28); (c) OCD (*n* = 23); (d) Anxiety disorders (GAD, PAD, SAD, Phobia: *n* = 16); (e) BPAD (*n* = 16)	

* Chi-square test.

GHQ: General Health Questionnaire; GAD: Generalized Anxiety Disorder; PAD: Panic Anxiety Disorder; SAD: Social Anxiety Disorder; OCD: Obsessive Compulsive Disorder; BPAD: Bipolar affective disorder.

### 2.2. Design

A cross-sectional study design was followed. The inclusion criteria were: (1) subjects aged 18-70 years who could read and write in English, Kannada, or Hindi; (2) Minimum educational qualification of 7th standard; (3) diagnosis of one of the following psychiatric disorders as per DSM-5 with mild to moderate severity of symptoms (only for patient population): anxiety (generalized anxiety disorder, panic anxiety disorder, social anxiety disorder, phobias), depression, obsessive compulsive disorder (OCD), bipolar affective disorder (BPAD), or schizophrenia (SCZ); and (4) healthy controls (HCs) with a general health questionnaire (GHQ-12) score ≤ 3. Exclusion criteria were those with: (1) other co-morbid neuropsychiatric disorders; (2) complete remission of clinical symptoms; (3) organic conditions, (4) protracted physical illness, (5) psychotic illness with severe symptoms, (6) cognitive decline (Mini mental status examination scale score < 23), or (7) intellectual disability.

Written informed consent was obtained from the participants and their caregivers. The study was approved by the National Institute of Mental Health and Neurosciences (NIMHANS) Institutional Ethics Committee (No. NIMHANS/EC(BEH.SC.DIV.)/11th Meeting/2018). The work described has been carried out in accordance with the Code of Ethics of the World Medical Association (Declaration of Helsinki) for experiments involving humans.

### 2.3. Instruments

#### 2.3.1. For patients

Sociodemographic proforma: This included the demographic details of the patients with psychiatric history, comorbid physical illnesses, and their mental status examination findings, and standard psychometric tools as described below.

##### 2.3.1.1. Hamilton depression rating scale (HAM-D)

The 17-item tool is the most widely used scale for controlled clinical trials in depression, with good reliability, validity, and internal consistency (Hamilton, 1960). Cut-off scores: no depression (0–7); mild depression (8–16); moderate depression (17–23); and severe depression (≥24).

##### 2.3.1.2. Hamilton anxiety rating scale (HAM-A)

The HAM-A is a 14-item, clinician-administered, semi-structured interview designed to assess anxiety symptoms not specific to any disorder. It has demonstrated excellent validity and reliability (Maier et al., 1988). The optimal HAM-A score ranges are: mild anxiety = 8–14; moderate = 15–23; severe ≥24 (scores ≤7 are considered to represent no/minimal anxiety).

##### 2.3.1.3. Yale-Brown obsessive–compulsive rating scale

This widely used 10-item scale rates the severity of obsessions and compulsions. Inter-rater reliability for the OCD severity score has been estimated at 0.95 (Goodman et al., 1989). Total Y-BOCS scores range from 0 to 40, with higher scores indicating greater severity of OCD symptoms. Y-BOCS score ranges are: subclinical (below 7), mild (8–15), moderate (16–23), severe (24–31) and extreme (32–40).

##### 2.3.1.4. Brief psychiatric rating scale

The BPRS covers 24 items across all psychosis symptom domains. The scale is sensitive to change and has good reliability and validity (Tarsitani et al., 2019). A BPRS score of 31 is considered as ‘mildly ill,’ a score of 41 is ‘moderately ill’, and 53 is ‘markedly ill’.

##### 2.3.1.5. Scale for assessment of positive symptoms

To assess positive symptoms in psychotic disorders, it consists of 34 items divided into four positive symptom subscales: hallucinations, delusions, bizarre behavior, and positive formal thought disorder (Charernboon, 2019). Clinical symptom cut-off score: 3 or above.

##### 2.3.1.6. Scale for assessment of negative symptoms

SANS measures negative symptoms and consists of 22 items divided into five subscales. A global score for each subscale intended to summarize all symptoms was also included (Dollfus et al., 2019). Clinical symptom cut-off score: 3 or above.

#### 2.3.2. For healthy controls

Socio-demographic Proforma: This included the demographic details with history and clinical examination findings to rule out co-morbidities as per the selection criteria.

##### 2.3.2.1. General health Questionnaire-12

The GHQ-12 is a self-reported screening tool with good reliability. It measures the current mental health status of an individual on a 4-point Likert scale, with a total score of 36 (Furnham and Cheng, 2019).

#### 2.3.3. For both patients and healthy controls

##### 2.3.3.1. Vedic personality inventory

*Guna* scores (*sattva*, *rajas*, *and tamas*) were assessed in all subjects (*n* = 226) using the VPI. VPI was devised by David Wolf to assess the validity of the *Vedic* concept of the three *gunas* or modes of nature, as a psychological categorization system (Wolf, 1999). The original 90-item VPI was shortened to 56 items based on reliability and validity analyses. Cronbach’s alpha for the three subscales ranged from.93 to.94, and the corrected item-total correlation of every item on the VPI with its subscale was greater than 0.50 (Stempel et al., 2006). While scoring, the standard score for each *guna* was calculated by dividing individual *guna* scores by the total score and multiplying it by 100. Thus, scores of *sattva* (S), *rajas* (R), and *tamas* (T) for each individual were obtained in percentage in such a way that their total (S + R + T) is 100%.

### 2.4. Data collection and statistical analyses

A psychiatrist applied the standard psychometric scales based on the diagnosis of the patient, whereas the VPI was filled by the participant using an online version of the scale. Help was sought, and responses were corroborated by caregiver while filling up VPI for patients with psychosis (who had BPRS score of 41 or above). Psychometric scales were applied first, followed by VPI. Both assessments were performed in the same session for all participants. We used R version 4.1.2 for analyzing the data. The R’s ggplot2 package was used to plot the distribution of data. Since there were more than two dependent variables, multivariate analysis of variance (MANOVA) was applied for analysis. Post-hoc analysis was performed using Scheffe’s test after age and gender correction using a generalized linear model to assess the differences in *guna* scores between HCs and patients, and between the patients with different psychiatric diagnoses (SCZ: *n* = 28, BPAD: *n* = 16, depression: *n* = 30, anxiety: *n* = 17, and OCD: *n* = 23). Examination of data revealed that all the assumptions for applying MANOVA were met. Two-tailed Pearson’s correlation was applied to correlate the scores of psychometric scales with *guna* scores of all groups of patients.

## 3. Results

### 3.1. Baseline clinical symptom scores and *Guna* scores

The recruited patients had mild to moderate severity of psychiatric illnesses. Table 2 provides the scores on standardized clinical rating scales for respective psychiatric diagnoses. Patients with anxiety had an average score of 15.34 ± 6.08 on HAM-A, those with depression scored 15.84 ± 5.52 on HAM-D, subjects with OCD had an average score of 20.48 ± 7.61 on Y-BOCS and subjects with psychotic disorders also displayed mild to moderate severity (average scores on BPRS, SAPS, and SANS were 48.71 ± 11.46,16.86 ± 6.31 and 17.18 ± 14.77, respectively). Healthy controls had an average GHQ-12 score of 1.1 ± 0.44.

**Table 2:** Psychiatric disorder with scores on respective standardized scale.

**SN**	**Psychiatric disorder**	**Average score on psychometric scale**
**1**	Anxiety disorder	15.34 ± 6.08 (HAM-A) (mild to moderate)
**2**	Depression	15.84 ± 5.52 (HAM-D) (mild to moderate)
**3**	Obsessive compulsive disorder	20.48 ± 7.61 (YBOCS) (moderate)
**4**	Schizophrenia and Bipolar affective disorder	16.86 ± 6.31 (SAPS); 17.18 ± 14.77 (SANS); 48.71 ± 11.46 (BPRS) (mild to moderate)
**5**	Healthy controls	1.1 ± 0.44 (GHQ-12)

HAM-A: Hamilton Anxiety Rating Scale; HAM-D: Hamilton Depression Rating Scale; YBOCS: Yale-Brown Obsessive–Compulsive Scale; SAPS: Scale for Assessment of Positive Symptoms; SANS: Scale for Assessment of Negative Symptoms; BPRS: Brief Psychiatric Rating Scale; GHQ: General Health Questionnaire.

The average *guna* scores for the healthy controls and patients with psychiatric disorders are listed in Table 3.

**Table 3:** Mean scores of Gunas in healthy controls and patients with psychiatric illnesses on Vedic Personality Inventory.

***Gunas***	**Healthy controls**	**Schizophrenia**	**Bipolar affective disorder**	**Depression**	**Anxiety disorder**	**Obsessive compulsive disorder**
***Sattva***	47.8 ± 8.5	27.5 ± 7.3	30.4 ± 7.5	38.4 ± 6.03	38.9 ± 4.8	41.9 ± 7.0
***Rajas***	29.2 ± 5.4	36.6 ± 4.6	32.8 ± 4.9	28.9 ± 4.9	32.9 ± 4.0	33.9 ± 3.0
***Tamas***	23.1 ± 5.3	35.9 ± 5.5	36.9 ± 4.5	32.7 ± 5.7	28.2 ± 3.2	25 ± 5.7

### 3.2. Between group comparisons

#### 3.2.1. Overall results – healthy controls versus patients:

HCs had significantly higher levels of *sattva* than patients with all other psychiatric disorders (*F*_(7, 234)_ = 35.07; *p* < 0.01), except for OCD (*F*_(7, 234)_ = 35.07; *p* = 0.09).

#### 3.2.2. Between patients with psychotic and non-psychotic disorders and healthy controls

It was observed that patients with psychotic disorders (SCZ and BPAD) had significantly lower levels of *sattva* (*F*_(7, 234)_ = 35.07; *p* < 0.05) and higher levels of *tamas* than patients with other psychiatric illnesses (*F*_(7, 234)_ = 37.87; *p* < 0.01) as well as HCs (*F*_(7, 234)_ = 37.87; *p* < 0.01). Patients with SCZ also had significantly higher levels of *rajas* than patients with depression (*F*_(7,234)_ = 11.64; *p* < 0.01) and HCs (*F*_(7, 234)_ = 11.64; *p* ≤ 0.01). *Tamas* scores did not significantly differ between patients with BPAD, SCZ, and depression, but all of them had higher *tamas* scores than patients with OCD (*F*_(7, 234)_ = 37.87; *p* < 0.01), and HCs (*F*_(7, 234)_ = 37.87; *p* < 0.01), respectively.

#### 3.2.3. Between patients with non-psychotic disorders and healthy controls

**Figure 1:**
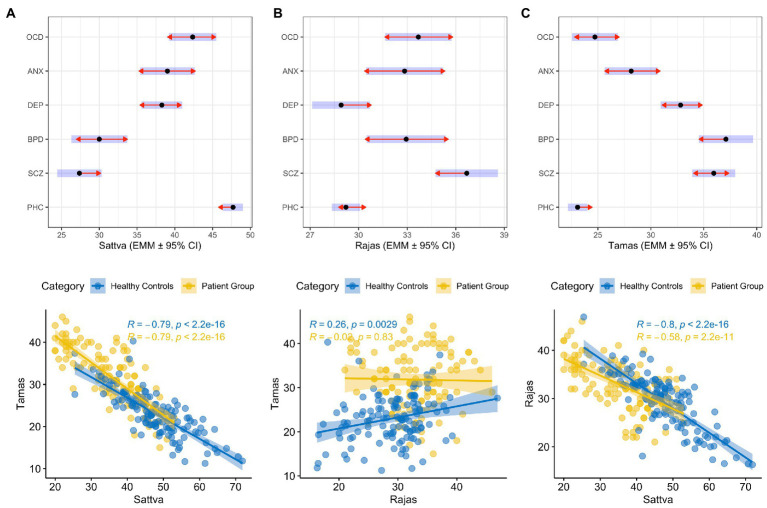
Distribution of **(A)** Sattva, **(B)** Rajas and **(C)** Tamas guna scores in patients with common psychiatric illnesses and healthy controls represented by Estimated Marginal Means ± 95% Confidence Interval (CI) shown with dot and blue bars. The red arrows are for the post-hoc Scheffe comparisons, with non-overlapping arrows indicating significant between-group differences. The panels D, E and F show correlation between gunas in patients and healthy controls. OCD: Obsessive-Compulsive Disorder; ANX: Anxiety Disorder; DEP: Depression; BPD: Bipolar affective Disorder; SCZ: Schizophrenia; PHC: Population Healthy control; R: Correlation coefficient.

Patients with anxiety had higher *rajas* scores (*F*_(7, 234)_ = 3.62; *p* ≤ 0.01) and lower tamas scores (*F*_(7, 234)_ = 5.05; *p* ≤ 0.01) than HCs. Patients with OCD had significantly higher levels of *rajas* than HCs (*F*_(7, 234)_ = 11.64; *p* ≤ 0.01) and patients with depression (*F*_(7,234)_ = 11.64; *p* = 0.03), respectively. Patients with depression had higher *tamas* scores than healthy controls (*F*_(7, 234)_ = 37.87; *p* < 0.01) and patients with OCD (*F*_(7, 234)_ = 37.87; *p* < 0.01). Table 4 provides details of the results obtained by comparing *guna* scores between healthy controls (HCs) and patients with different categories of psychiatric disorders. The distribution of scores of the three *gunas* (*sattva*, *rajas*, and *tamas*) across common psychiatric disorders and healthy controls are displayed in Figures 1A–C.

**Table 4:** Comparison for different gunas between healthy controls and patients suffering from psychiatric disorders.

	*Sattva*				*Rajas*				*Tamas*			
Predictors	Estimates	SE	Statistic	*p*	Estimates	SE	Statistic	*p*	Estimates	SE	Statistic	*p*
Intercept	41.38	1.74	23.77	**<0.001**	32.26	1.15	28.16	**<0.001**	26.60	1.20	22.09	**<0.001**
Age (y)	0.18	0.05	3.39	**0.001**	−0.08	0.03	−2.47	**0.014**	−0.10	0.04	−2.75	**0.007**
Gender (Female vs. Male)	1.86	1.02	1.83	0.068	−0.94	0.67	−1.41	0.161	−0.99	0.70	−1.41	0.160
Gp (SCZ vs. PHC)	−20.34	1.65	−12.31	**<0.001**	7.47	1.09	6.87	**<0.001**	12.85	1.14	11.25	**<0.001**
Gp (BPD vs. PHC)	−17.71	1.99	−8.89	**<0.001**	3.72	1.31	2.84	**0.005**	14.00	1.38	10.16	**<0.001**
Gp (DEP vs. PHC)	−9.42	1.54	−6.10	**<0.001**	−0.30	1.02	−0.29	0.771	9.71	1.07	9.10	**<0.001**
Gp (ANX vs. PHC)	−8.68	1.97	−4.41	**<0.001**	3.62	1.30	2.80	**0.006**	5.05	1.36	3.71	**<0.001**
Gp (OCD vs. PHC)	−5.34	1.74	−3.08	0.09	4.47	1.14	3.92	**<0.001**	1.63	1.20	1.36	0.176
Observations	242				242				242			
R^2^ / R^2^adjusted*	0.512 / 0.497				0.258 / 0.236				0.531 / 0.517			

* Scheffe’s test after age and gender correction using generalized linear model. Bold values represent statistically significant differences.

### 3.3. Correlations

**Figure 2:**
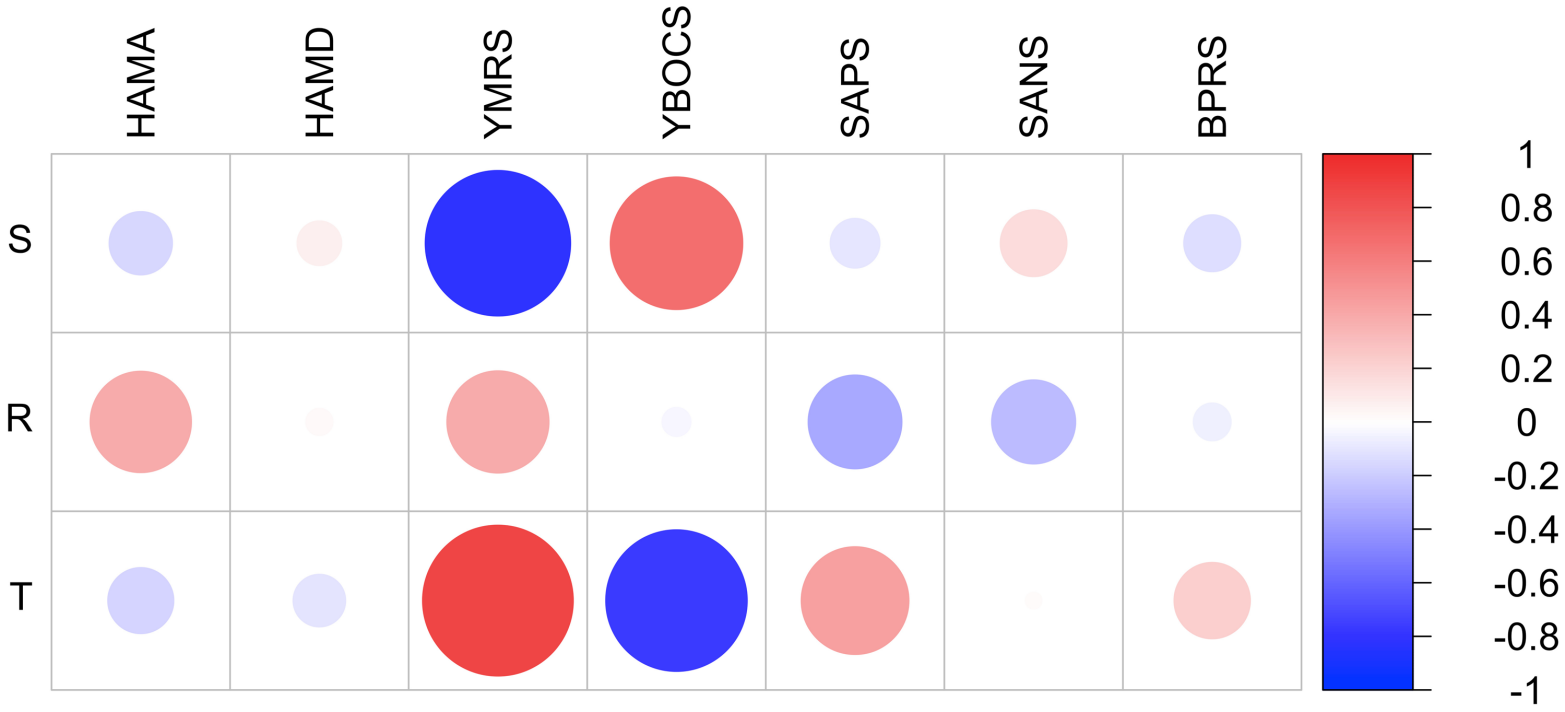
Corplot showing correlation between the gunas and standard psychometric scales in patients with psychiatric illnesses. HAM-A: Hamilton Anxiety Rating Scale: HAM-D; Hamilton Depression Rating Scale; YBOCS: Yale-Brown Obsessive–Compulsive Scale; SAPS: Scale for Assessment of Positive Symptoms; SANS: Scale for Assessment of Negative Symptoms; BPRS: Brief Psychiatric Rating Scale; YMRS (Young Mania Rating Scale); GHQ: General Health Questionnaire; S: Sattva; R: Rajas; T: Tamas. Scale on Y-axis denotes correlation coefficient values. Colour bar denotes correlation coefficient values.

Two-tailed Pearson’s correlation revealed significant positive correlations between the following scales: (1) *Rajas scores* and anxiety symptoms (HAM-A, *r* = 0.31; *p* = 0.02); *rajas* and obsessive–compulsive symptoms (Y-BOCS, *r* = 0.46; *p* = 0.03); *rajas* and positive psychotic symptoms (SAPS, *r* = 0.56; *p* = 0.01); (2) *Sattva* scores and obsessive–compulsive symptoms (Y-BOCS, *r* = 0.55; *p* = 0.01); and (3) *Tamas* scores and negative psychotic symptoms (SANS, *r* = 0.77; *p* = 0.01). A significant negative correlation was noted between obsessive–compulsive symptoms and *tamas* scores (Y-BOCS, *r* = −0.83; *p* = 0.01), and anxiety symptoms and *sattva* scores (HAM-A, *r* = −0.30; *p* = 0.02) (Table 5). Furthermore, negative correlations were found between *sattva* and *tamas* (*r* = −0.79; *p* < 0.01) and *sattva* and *rajas* in patients (*r* = −0.58; *p* < 0.01) and HCs (*r* = −0.8; *p* < 0.01). Interestingly, a positive correlation between *rajas* and *tamas* was observed in HCs (*r* = 0.26; *p* = 0.002) but not in patients (*r* = −0.02; *p* = 0.83; Table 5). Figure 1 provides a graph depicting the correlation between the *gunas* for patients and population healthy controls (PHC). We also observed a negative correlation of Young Mania Rating Scale (YMRS) with *sattva* scores (*r* = -0.8; *p* < 0.01) and positive correlation of the same with *tamas* scores (*r* = 0.74; *p* < 0.01) but sample size of patients with manic symptoms was very low (*n* = 7). Figure 2 provides a correlation plot showing the relationship between the three *gunas* and various psychiatric clinical rating scales.

**Table 5:** Significant statistical correlation between psychometric scales and S,R,T factors.

**SN**	**Psychometric scale**	***Guna*s**	**R value**	***P***^**a**^ **value**
**1**	HAM-A	*Rajas*	+0.31	0.02*
**2**	Y-BOCS		+0.46	0.03*
**3**	SAPS		+0.56	0.01**
**4**	HAM-A	*Sattva*	−0.30	0.02*
**5**	Y-BOCS		+0.55	0.01**
**6**	Y-BOCS	*Tamas*	−0.83	0.01**
**7**	SANS		+0.77	0.01**

a Pearson’s two-tailed correlation. *p < 0.05 **p<0.01.

HAM-A: Hamilton Anxiety Rating Scale; HAM-D: Hamilton Depression Rating Scale; YBOCS: Yale-Brown Obsessive–Compulsive Scale; SAPS: Scale for Assessment of Positive Symptoms; SANS: Scale for Assessment of Negative Symptoms; BPRS: Brief Psychiatric Rating Scale; GHQ: General Health Questionnaire.

## 4. Discussion

In this cross-sectional trans-diagnostic study comparing patients suffering from common psychiatric disorders with age-gender-education matched healthy subjects, we observed that healthy controls had significantly higher *sattva guna* scores compared to patients suffering from psychiatric disorders with mild to moderate illness. We also observed a different pattern of *guna* distribution in different categories of psychiatric disorders (Table 3): (1) Anxiety disorders and OCD: High *sattva-rajas*, low *tamas*; (2) Depression: High *sattva-tamas*, low *rajas;* (3) Psychotic disorders (Schizophrenia/BPAD): High *tamo-rajas*, low *sattva*. In addition, significant positive correlations were observed between (1) *rajas* traits and anxiety/OC/positive psychotic symptoms, (2) *sattva* traits and OC symptoms, and (3) negative psychotic symptoms and *tamas* traits. All the above findings are in line with our hypothesis. The only result that is not as per the hypothesis is no significant correlation between depressive symptoms and tamas traits. This understanding of psychiatric disorders as per the *guna* perspective is relevant to Indian culture and traditions. For example, traditional yogic texts that emphasize *Samkhya* school of philosophy (such as *Bhagavad Gita* or *Patanjali’s Yoga Sutra*) specifically categorize mind and its modifications based on *guna* configurations (P.Y.S. 2.19, B.G. 18.19). Interestingly, yogic texts also provide specific techniques of yoga and lifestyle management strategies such as diet, physical activity, recreation and sleeping patterns to target *guna* modifications in a particular direction (B.G. 6.17). For example, the approach to manage a patient with depression from Indian traditional perspective would be to first understand the *guna* configuration. Based on the current findings the guna configuration in depression reveals high *sattva* and *tamas* scores, and low *rajas* scores. Thus, the techniques of yoga and lifestyle advices would focus on reducing *tamas* and enhancing *rajas* traits. This would include practices such as dynamic sun salutations (*Aditya Hridaya Stotra*), exposure to sun light, regular walks two times a day, right nostril (sun-channel) breathing (H.Y.P. 2.50), chanting of the mantra with sound “AAA” (*Mandukya Upanishad* verse 9) or *Gayatri* mantra, avoiding daytime sleep, meditation on *Manipura chakra* (solar energy centre at the navel region) or “fire” principle (*Shiva samhita* chapter 5, verse 81), or imageries focusing on “Sun” and its energy (*Aditya Hridaya Stotra*), diet that enhances digestive fire and activates the mind (emphasis on bitter, sour, salty, spicy tastes and consuming food that is hot and dry in nature and enhances digestive fire; B.G. 17.9). Such patients may also be advised to undergo yogic-psychotherapy for transcendence from *sattva* trait to *Gunatita* trait, especially if *sattva* scores are high (B.G. 14.22). In this way, specific lifestyle changes can be advised in different psychiatric conditions.

A previous study compared 30 healthy subjects with 30 patients suffering from anxiety disorders and found that patients with anxiety disorder had higher *rajas* and *tamas* scores and poorer quality of life than healthy subjects (Sharma et al., 2012). It was also observed that the quality of life positively correlated with higher *sattva* scores in healthy subjects, whereas higher *rajas* and *tamas* scores found in the clinical population were associated with reduced quality of life. In the current study, we observed higher *rajas* scores and lower *tamas* scores in anxiety patients as compared to HCs. We also observed a positive correlation between HAM-A and *rajas* scores. This suggests that a *rajas* personality trait may predispose a person to developing anxiety states. However, in a cross-sectional study like this one should be cautious about making a causal inference, it is also possible that anxiety would increase the *rajas* trait. We did not observe a significant correlation between HAM-A and *tamas* scores, as in a previous study. This may be explained as follows: as per yoga text (Bhagavad Gita, 14.8), the description of *tamas* indicates traits that are not generally seen in patients with anxiety. In fact, a general clinical observation is that people with anxiety disorders have more tendencies toward *rajas* traits than *tamas* traits; second, the clinical population involved in our study belonged to the category of mild anxiety (HAM-A mean ± SD: 15.34 ± 6.08). It is quite possible that patients with chronic and severe anxiety may develop *tamas* tendencies over a period of time, which may manifest in the form of lack of productivity and mental dullness. Thus, the underlying *tamas guna*, which may be evident in more severe cases, may not have manifested in the current population. It would be interesting to assess the *guna* profiles across different severity levels within psychiatric diagnoses in future studies.

Another preliminary investigation reported higher *rajas* scores in depressed subjects (*n* = 20) than in healthy individuals (*n* = 20; Anoop Kumar and Balodhi, 2016). In the current study, we did not observe any significant relationship between HAM-D and *guna* scores in 30 subjects. This may be because the patient population enrolled in our study had depression scores in the mild to moderate range (HAM-D = 15.84 ± 5.52). Even then, the subjects in this subgroup showed the lowest scores on *rajas* (28.9 ± 4.93) as compared to the other two *gunas; sattva* (38.4 ± 6.03) and *tamas* (32.7 ± 5.76). Although this is not consistent with earlier research findings, it is in accordance with the description of *rajas* in ancient yogic texts. The *rajas* traits of ambitiousness and attachment to action do not reflect the symptoms of depression where the subject feels hopeless, dejected, and less inclined toward activity. At face value, it appears that a depressed subject should show a higher *tamas* trait, but we observed that their scores were higher on *sattva* trait than *tamas*. Sometimes high levels of *sattva* may make an individual emotionally sensitive and rigid in perfectionism and fairness. Any failure to live up to it may push him/her into depression. A larger sample size may be required in future studies to obtain conclusive findings. It would also be interesting to understand *the guna* profiles of subjects with moderate and severe levels of depression in future studies.

We found another small study on subjects with psychotic disorders. In this study, *guna* scores of 15 patients with different psychotic disorders were compared with 30 healthy subjects on their *guna* scores (Lakshmi Bai et al., 1975). The study reported that psychotic patients had higher *rajas* and *tamas* scores than the normal population, which was also observed in the current study. Aggressiveness, agitation, grandiose ideas, and distress due to hallucinations and delusions may have resulted in such a correlation with *rajas guna*. Negative symptoms of psychosis, such as apathy and social withdrawal suggesting inertia, might have translated to a positive correlation between SANS scores and *tamas*.

To the best of our knowledge, no previous study has reported *the guna* profiles of subjects suffering from OCD. We observed that *sattva* scores were not significantly different in patients with OCD than HCs. Furthermore, patients had the highest *sattva* scores compared to all other psychiatric disorders. It was observed that YBOCS scores were positively correlated with *sattva* and negatively correlated with *tamas* scores (Figure 1). The *sattva guna* may underlie the excess sense of morality and responsibility appraisal that is found in patients with OCD (O’Leary et al., 2009). In addition, an excess of the *sattva guna* may lead to intolerance towards unrighteousness, either in the mental or physical aspects of a person’s life. This predisposes *sattva* dominant individuals to intolerance towards unpleasant and unconducive situations. A tendency to strive for perfection and stability may be a basic substrate underlying *sattva guna* and obsessive–compulsive tendencies, respectively. The negative correlation of YBOCS with *tamas* may indicate an innate trait of perfectionism in patients who develop OCD, in conflict with the psychological inertia of *tamas*. It is also possible that obsessions and compulsions may serve a compensatory effect on cognitive inflexibility and inertia (Ramakrishnan et al., 2022). Thus, clinically, a patient with OCD would benefit from a lifestyle program that takes an individual beyond the limitations of *sattva traits* towards the transcendental *(gunatita)* traits. In the current study, negative correlations were observed between *sattva* and *tamas*, and *sattva* and *rajas* in patients and HCs, respectively (Figure 1). According to yogic texts, these three *gunas* exist as inherent components of every psyche and serve as the binding forces that bind the consciousness (*purusha*) to the body (B.G. 14.5), when one trait manifests, the other two are suppressed (B.G. 14.10). Interestingly, a positive correlation between *rajas* and *tamas* was observed in HCs, but not in patients (Figure 1). This might be due to the proper reciprocal functioning of *rajas* and *tamas gunas* in healthy individuals in order to maintain equanimity. This form of reciprocal functioning of *tamas* and *rajas* seems to be deranged in psychiatric disorders as evident from the *guna* scores reported in this study (Figure 1). This may also be related to a ceiling effect in patients, where both *rajas* and *tamas* features are elevated, and therefore reducing variance.

The nature of *gunas* is viewed as dynamic in yoga-philosophical concepts, as these concepts account for both the state and trait features of a disorder. This can also explain certain life-changing experiences that produce changes in personality, substantiating the plasticity of personality traits. Traditional yoga texts provide a systematic lifestyle plan for various *guna-*based personality types that can promote transition from one *guna* to another over a period of time. For example, as discussed above, there are specific dietary prescriptions and yogic practices mentioned in yoga texts that promote the prognostic transitions of psychological tendencies from *tamas* to *rajas,* and *rajas* to *sattva*, and *sattva* to *gunatita* (B.G. 14.9–14.27; B.G. 17.8–17.10; Gambhirananda, 1984; Lauricella, 2021). These lifestyle suggestions as per the *guna-based* diagnosis, could potentially help patients in improving not only the clinical status, but also overall well-being.

The strengths of the current study include the multi-disciplinary trans-diagnostic approach, use of larger number of healthy controls to understand the deflections in *gunas* specifically in each psychiatric disorder, use of scientifically validated tools, inclusion of major psychiatric disorders, and exploration of ancient *vedic* knowledge. Limitations of the study include the cross-sectional nature of the study, inclusion of patients attending a single hospital, relatively lesser number of subjects under each psychiatric diagnosis, and lack of variety in the severity of diseases.

This study has important implications for future research. Yoga therapy in the current form utilizes only a set of practices at the body, breath, and mind levels. However, the psychotherapeutic potential of yoga-philosophy has not been explored. Also, the underlying philosophy related to the human psyche is not considered while designing such yoga modules. Understanding the psyche and psychopathological state of an individual from traditional perspective may facilitate the incorporation of specific yogic psychotherapeutic and lifestyle interventions (modifying bio-rhythms and diet) for prognostic modifications of *gunas* leading to wellbeing. Future studies should replicate these findings in large number of psychiatric patients and include patients with different levels of severity. It is crucial that future studies aim to investigate longitudinal trajectories of the *gunas* in health and disease. This will help understand the direction of association between *gunas* and clinical symptoms, i.e., whether *gunas* predispose a person to certain psychiatric disorders or vice versa. Future studies should also develop, validate, and test the efficacy of *guna*-based psychotherapeutic and lifestyle interventions in psychiatric patients and assess whether this translates into improved clinical outcomes.

## 5. Conclusion

Yoga-philosophy based mental traits *(Guna)* scores could differentiate healthy subjects from those with psychiatric disorders. *Guna* traits also varied between different psychiatric disorders. This may help provide a basis for developing psychotherapeutic and lifestyle modification programs based on the patient’s *guna* profile. It may also help develop ways of predicting outcomes of psychiatric disorders.

## Data availability statement

The raw data supporting the conclusions of this article will be made available by the authors, without undue reservation.

## Ethics statement

The studies involving human participants were reviewed and approved by Nimhans Human Behavioral Sciences Ethics Committee. The patients/participants provided their written informed consent to participate in this study.

## Author contributions

HB and BG conceptualized the idea. NE and HB designed the study and drafted the manuscript. NJ, HB, PM, and VK helped in data collection. BH analyzed the data. NR, RA, SV, BG, and MK reviewed and edited the manuscript. All authors contributed to the article and approved the submitted version.

## Funding

HB is supported by DBT/Wellcome Trust India Alliance Early Career Fellowship, Grant/Award Number: IA/CPHE/21/1/505978. SV is supported by DBT/Wellcome Trust India Alliance Intermediate Career Fellowship. Authors also acknowledge the support from Central Council for Research in Yoga & Naturopathy (CCRYN) grant from the Ministry of AYUSH, Government of India (Grant No: 002/208/2016/00925/CCRYN/CRC/NIMHANS).

## Conflict of interest

The authors declare that the research was conducted in the absence of any commercial or financial relationships that could be construed as a potential conflict of interest.

## Publisher’s note

All claims expressed in this article are solely those of the authors and do not necessarily represent those of their affiliated organizations, or those of the publisher, the editors and the reviewers. Any product that may be evaluated in this article, or claim that may be made by its manufacturer, is not guaranteed or endorsed by the publisher.
